# Efficacy and safety of tenofovir amibufenamide in patients with chronic hepatitis B: a real-world clinical study

**DOI:** 10.3389/fmed.2026.1774942

**Published:** 2026-02-26

**Authors:** Rongshan Fan, Hongmei Fan, Linling Tan, Jun Zhang, Li Ai

**Affiliations:** Department of Hepatology, Shenzhen Hospital of Integrated Traditional Chinese and Western Medicine, Shenzhen, Guangdong, China

**Keywords:** chronic hepatitis B, efficacy, real-world study, safety, tenofovir amibufenamide

## Abstract

**Objective:**

To assess the real-world efficacy and safety of tenofovir amibufenamide (TMF) in patients with chronic hepatitis B (CHB) and to generate evidence to inform optimization of antiviral treatment selection in routine clinical practice.

**Methods:**

This study enrolled 186 patients with CHB, of whom 93 received TMF and 93 received tenofovir disoproxil fumarate (TDF) for 48 weeks. The primary endpoint was the virological response rate. Secondary endpoints included the rate of serum alanine aminotransferase (ALT) normalization; changes in hepatitis B surface antigen (HBsAg) and hepatitis B e antigen (HBeAg) levels; and changes in renal and bone metabolism markers, including serum creatinine, uric acid, calcium, and phosphate.

**Results:**

After 48 weeks of treatment, virological response rates did not differ significantly between the TMF and TDF groups (*p* > 0.05). In contrast, the ALT normalization rate was significantly higher in the TMF group than in the TDF group (76.34% vs. 60.22%, *p* < 0.05). Similarly, the HBeAg seroconversion rate was markedly higher with TMF therapy (22.78% vs. 9.21%, *p* < 0.05). With respect to safety, serum creatinine levels at week 48 were significantly lower in the TMF group compared with the TDF group (77.36 ± 16.87 μmol/L vs. 90.12 ± 17.23 μmol/L, *p* < 0.05). No significant between-group differences were observed in serum uric acid, calcium, or phosphate levels.

**Conclusion:**

In real-world clinical practice, TMF is an effective treatment for CHB. Compared with TDF, TMF is associated with superior ALT normalization and higher HBeAg seroconversion rates, along with a more favorable renal safety profile.

## 1 Introduction

Chronic infection with hepatitis B virus (HBV) imposes a substantial global disease burden. An estimated 257.5 million individuals worldwide are chronically infected with HBV, and approximately 900,000 deaths occur annually due to end-stage liver diseases, including liver failure, decompensated cirrhosis, and hepatocellular carcinoma (1, 2). Sustained suppression of HBV replication remains the cornerstone of treatment for chronic hepatitis B. Currently, first-line oral antiviral agents widely used in China include entecavir (ETV), tenofovir disoproxil fumarate (TDF), tenofovir alafenamide fumarate (TAF), and tenofovir amibufenamide (TMF) (3). Phase III clinical trials have demonstrated that TMF provides potent antiviral efficacy with favorable bone and renal safety profiles (4). However, real-world evidence regarding the effectiveness and safety of TMF in patients with chronic hepatitis B remains limited. Therefore, this study aimed to evaluate the real-world efficacy and safety of TMF in the treatment of chronic hepatitis B, to delineate its clinical advantages and limitations, and to inform the selection of antiviral treatment strategies.

## 2 Materials and methods

### 2.1 Case selection

This retrospective randomized cohort study included 186 patients with chronic hepatitis B who were treated at Shenzhen Hospital of Integrated Traditional Chinese and Western Medicine between June 2024 and September 2025. Among them, 93 patients received TMF (25 mg/day) for 48 weeks, and 93 patients received TDF (300 mg/day) for the same duration. The cohort comprised 119 men and 67 women, with ages ranging from 23 to 62 years and a mean age of 37.96 ± 7.56 years.

The inclusion criteria were as follows: newly diagnosed chronic hepatitis B; fulfillment of the diagnostic criteria outlined in the Guidelines for the Prevention and Treatment of Chronic Hepatitis B (2022 edition) (3); serum HBV DNA ≥ 20 IU/mL; positive serum hepatitis B surface antigen (HBsAg); disease duration exceeding 6 months; and good treatment compliance.

Exclusion criteria included liver failure; co-infection with hepatitis C virus or human immunodeficiency virus; autoimmune liver disease; heart failure, respiratory failure, malignancies, or central nervous system disorders; liver cirrhosis; hepatocellular carcinoma; and pregnancy.

### 2.2 Research methods

Patients receiving TMF were assigned to the TMF group, whereas those treated with TDF constituted the TDF group. The primary outcome was the virological response rate, defined as an HBV DNA level <20 IU/mL at week 48 of treatment. Secondary outcomes included changes in serum alanine aminotransferase (ALT), HBsAg, hepatitis B e antigen (HBeAg), serum creatinine, uric acid, calcium, and phosphate levels at week 48, as well as the virological response rate in patients with a high viral load. High viral load was defined as a baseline HBV DNA concentration >1.0 × 10^7^ IU/mL.

### 2.3 Detection method

Biochemical parameters were measured using a fully automated biochemical analyzer. The reference range for ALT was 9–50 U/L in men and 7–40 U/L in women. The five serological markers of hepatitis B were assessed using electrochemiluminescence assays, and quantitative HBV DNA levels were determined by fluorescence-based real-time polymerase chain reaction.

### 2.4 Statistical analysis

Statistical analyses were performed using SPSS version 26.0. To facilitate analysis, HBV DNA, HBsAg, and HBeAg values were logarithmically transformed. Data normality was assessed using the Shapiro–Wilk test, and homogeneity of variance was evaluated with the Levene test. Normally distributed data with homogeneous variance were expressed as mean ± standard deviation (x ± s), and compared using independent-samples *t* tests. Non-normally distributed data or data with unequal variance were reported as medians (interquartile ranges) and analyzed using rank-sum tests. Categorical variables were compared using the chi-square test. A *p* value <0.05 was considered statistically significant.

## 3 Results

### 3.1 Characteristics of the study subjects

A total of 186 patients were included in the final analysis, with 93 patients in the TMF group and 93 patients in the TDF group. No significant differences were observed between the two groups with respect to age, sex, body mass index, baseline HBsAg levels, HBeAg status, HBV DNA levels, or biochemical parameters (*p* > 0.05), indicating good baseline comparability. Detailed baseline characteristics are presented in Table 1.

**Table 1:** Characteristics of baseline/week 48 in the TMF and TDF groups (mean ± standard deviation).

Items	Time	TMF group (*n* = 93)	TDF group (*n* = 93)	Statistical value	*p* value
Age (years)	week 0	38.11 ± 7.23	37.82 ± 7.27	*t* = −1.32	>0.05
Male, *n* (%)	week 0	61 (65.59)	58 (62.37)	*X^2^* = 0.20	>0.05
BMI (kg/m^2^)	week 0	23.72 ± 2.93	23.47 ± 3.13	χ^2^ = 0.24	>0.05
HBeAg-positive, *n* (%)	week 0	79 (84.95)	76 (81.72)	χ^2^ = 0.34	>0.05
ALT (U/L)	week 0	61.22 ± 45.81	58.36 ± 42.66	*Z* = 0.51	>0.05
	week 48	34.28 ± 25.43*	32.82 ± 23.73*	*Z* = 0.86	>0.05
HBsAg (log_10_ IU/mL)	week 0	3.82 ± 0.91	3.75 ± 0.87	*Z* = 0.64	>0.05
	week 48	3.61 ± 0.79	3.65 ± 0.83	*Z* = 0.42	>0.05
HBeAg (log_10_ PEIU/mL)	week 0	1.92 ± 0.75	1.87 ± 0.81	*Z* = 0.58	>0.05
	week 48	1.52 ± 0.61*	1.59 ± 0.65*	*Z* = 0.93	>0.05
HBV DNA (log_10_ IU/mL)	week 0	5.81 ± 1.82	5.67 ± 1.94	*Z* = 0.75	>0.05
	week 48	1.92 ± 1.25*	2.14 ± 1.43*	*Z* = 1.18	>0.05
SCr (μmol/L)	week 0	76.84 ± 16.11	75.24 ± 15.36	*t* = −0.72	>0.05
	week 48	77.36 ± 16.87	90.12 ± 17.23	*t* = 13.78	<0.05
TG (mmol/L)	week 0	1.24 ± 0.68	1.31 ± 0.72	*Z* = 0.83	>0.05
	week 48	1.21 ± 0.69	1.27 ± 0.73	*Z* = 0.59	>0.05
TC (mmol/L)	week 0	4.52 ± 0.87	4.61 ± 0.92	*t* = 0.72	>0.05
	week 48	4.51 ± 0.95	4.58 ± 0.97	*t* = 0.53	>0.05
Uric acid (μmol/L)	week 0	298 ± 102	305 ± 98	*t* = 0.51	>0.05
	week 48	291 ± 105	297 ± 101	*t* = 0.43	>0.05
Serum calcium (mmol/L)	week 0	2.27 ± 0.53	2.32 ± 0.79	*t* = 0.69	>0.05
	week 48	2.25 ± 0.65	2.15 ± 0.58	*t* = 0.78	>0.05
Serum phosphate (mmol/L)	week 0	0.94 ± 0.31	1.13 ± 0.37	*t* = 0.42	>0.05
	week 48	1.18 ± 0.37	1.28 ± 0.47	*t* = 0.43	>0.05

ALT, alanine transaminase; BMI, body mass index; HBeAg, hepatitis B e-antigen; HBsAg, hepatitis B surface antigen; HBV, hepatitis B virus; SCr, serum creatinine; TC, total cholesterol; TG, triglyceride. ^*^ A significant difference was observed in the values between week 0 and week 48 in the same group (*p* < 0.05).

### 3.2 Comparison of therapeutic effects

At 48 weeks, the virological response rates were 78.49% in the TMF group and 83.87% in the TDF group, with no statistically significant difference between the two treatments (*p* > 0.05). Intragroup analyses demonstrated that HBV DNA levels decreased significantly from baseline in both groups (*p* < 0.05).

Among patients with high baseline viral loads, the virological response rates at week 48 were 62.50% in the TMF group and 58.82% in the TDF group, again without a significant intergroup difference (*p* > 0.05).

At week 48, the ALT normalization rate was significantly higher in the TMF group than in the TDF group (76.34% vs. 60.22%, *p* < 0.05). However, mean serum ALT levels were comparable between the TMF and TDF groups (34.28 ± 25.43 U/L vs. 32.82 ± 23.73 U/L, respectively; *p* > 0.05). Within-group comparisons revealed significant reductions in ALT levels from baseline to week 48 in both groups (*p* < 0.05).

No patients in either group achieved HBsAg loss at 48 weeks.

In contrast, the HBeAg seroconversion rate was significantly higher in the TMF group than in the TDF group (22.78% vs. 9.21%, *p* < 0.05). Intragroup analyses further indicated significant declines in HBeAg levels in both treatment groups by week 48 (*p* < 0.05).

### 3.3 Comparison of safety indicators

With respect to safety, serum creatinine levels at week 48 were significantly lower in the TMF group than in the TDF group (77.36 ± 16.87 μmol/L vs. 90.12 ± 17.23 μmol/L, *p* < 0.05), and an increasing trend in creatinine was observed in the TDF group. No significant differences were detected between groups in serum uric acid, triglycerides, total cholesterol, calcium, or phosphate levels at week 48 (all *p* > 0.05).

Detailed results are presented in Table 2.

**Table 2:** Comparison of the therapeutic effects between the groups.

Items	Time	TMF group (*n* = 93)	TDF group (*n* = 93)	Statistical value	*p* value
Ratio of ALT normalization (%)	week 48	71/93 (76.34)	56/93 (60.22)	*X^2^* = 5.585	<0.05
Ratio of virological response (%)	week 48	73/93 (78.49)	78/93 (83.87)	*X^2^* = 0.927	>0.05
Ratio of the virological response of patients with a high viral load (%)	week 48	15/24 (62.50)	20/34 (58.82)	*X^2^* = 0.079	>0.05
Ratio of HBeAg serum conversion (%)	week 48	18/79 (22.78)	7/76 (9.21)	*X^2^* = 5.347	<0.05

ALT, alanine transaminase; HBeAg, hepatitis B e-antigen.

## 4 Discussion

This real-world study evaluated the efficacy and safety of TMF and TDF in patients with chronic hepatitis B (CHB).

After 48 weeks of treatment, TMF demonstrated a significantly higher ALT normalization rate than TDF, indicating superior biochemical efficacy. Although serum ALT levels declined significantly in both groups, the greater normalization rate observed with TMF is clinically meaningful. Previous studies have shown that ALT levels, even when below the upper limit of normal, are closely associated with significant hepatic inflammatory necrosis and fibrosis (5, 6), as well as with adverse clinical outcomes such as decompensated cirrhosis (e.g., ascites and hepatic encephalopathy) and hepatocellular carcinoma (HCC) (7). The present findings suggest that TMF-induced reductions in ALT may contribute to alleviating hepatic inflammation and fibrosis, thereby potentially lowering the risk of cirrhosis and HCC. Of course, to further clarify the benefits of the decrease in ALT, future research should include liver biopsies or non-invasive tests, as well as long-term clinical outcomes.

At week 48, TMF and TDF achieved comparable virological response rates. The virological response rate of 78.49% observed with TMF was consistent with previously reported findings (8). Both agents effectively reduced HBV DNA levels, although viral load decline appeared more rapid in the TMF group. In patients with high baseline viral loads, the virological response rates to TMF and TDF were similar at week 48, in agreement with prior studies (9). HBeAg-positive patients with CHB are particularly susceptible to low-level viremia during nucleos(t)ide analog therapy due to high baseline viral replication. While previous evidence suggests that entecavir has limited efficacy and a higher incidence of low-level viremia in this population, TDF has demonstrated favorable antiviral activity in patients with high viral loads. The present study further indicates that TMF is effective in enhancing virological response in CHB patients with high viral loads.

In terms of serological outcomes, both TMF and TDF reduced HBsAg levels, although neither treatment achieved HBsAg clearance within the 48-week observation period. Both regimens induced HBeAg seroconversion, with TMF showing a significantly higher rate of HBeAg loss and seroconversion than TDF. By week 48, HBeAg levels had declined in both groups, with a more pronounced reduction observed in the TMF group. Data from non–head-to-head studies have reported variable HBeAg seroconversion rates after 5 years of nucleos(t)ide analog therapy (68% for TMF, 46% for TDF, and 54% for ETV) (10, 11). Although limited by the relatively short treatment duration, this 48-week study provides evidence supporting the superiority of TMF over TDF in promoting HBeAg decline and serological response.

The results of this study indicate that neither drug caused clinically significant renal injury during the treatment period. However, serum creatinine levels exhibited an upward trend at 48 weeks in patients receiving TDF. In contrast, creatinine levels in the TMF group remained relatively stable after 48 weeks and did not show a progressive increase. These findings suggest that TMF has a more favorable renal safety profile and is superior to TDF with respect to kidney protection, consistent with previous clinical studies (12). One possible explanation is the limited targeting efficiency of TDF: a daily dose of 300 mg is required to achieve effective intracellular concentrations in hepatocytes, while the resulting tenofovir accumulates extensively and persistently in plasma, potentially leading to nephrotoxicity. By comparison, TMF is administered at a lower dose, which may reduce systemic exposure and thereby mitigate renal damage (13).

TAF has been reported to affect lipid metabolism (14). Given the structural similarity between TMF and TAF, the potential impact of TMF on lipid metabolism has become an important concern. In this study, no significant dyslipidemia was observed during 48 weeks of TMF treatment. Whether longer-term TMF therapy may induce lipid abnormalities warrants further investigation. No abnormalities in serum calcium or phosphorus metabolism were detected during the observation period. However, bone mineral density was not assessed, and thus comprehensive conclusions regarding bone health cannot be drawn.

In conclusion, this real-world clinical study is different from the Phase III study of TMF (15). The research subjects did not limit the levels of ALT and HBVDNA, and did not rule out comorbidities such as hypertension, coronary heart disease, diabetes and fatty liver. It is a true reflection of clinical practice. This study demonstrates that TMF achieves virological response rates comparable to those of TDF. Moreover, TMF offers several advantages, including a higher rate of transaminase normalization, more rapid declines in HBeAg levels, higher HBeAg seroconversion rates, and improved renal safety profiles. Nonetheless, given the relatively short follow-up duration and limited sample size, continued random selection, multicenter research, longitudinal monitoring and larger-scale studies are required to more fully evaluate the long-term efficacy and safety of TMF. For special groups such as children, the elderly, pregnant and postpartum women, and patients with renal insufficiency, the safety of TMF also requires further observation.

## Data Availability

The original contributions presented in the study are included in the article/supplementary material, further inquiries can be directed to the corresponding author.
